# Comparison of different characterization approaches for monoelemental calibration solutions at two national metrology institutes

**DOI:** 10.1007/s00216-025-05731-4

**Published:** 2025-01-16

**Authors:** Murat Tunç, Cristhian Paredes, F. Gonca Coşkun, Juliana Serna, Merve Caner

**Affiliations:** 1 TÜBİTAK-UME National Metrology Institute, Gebze Yerleşkesi Barış Mah. Dr. Zeki Acar Cad. No:1, 41470, Gebze, Kocaeli Türkiye; 2 Grupo de Investigación en Metrología Química y Bioanálisis, Instituto Nacional de Metrología de Colombia, Ak. 50 26 - 55 Int. 2, Bogotá, 111321 Colombia; 3 Turkish Petroleum Refineries Co. TÜPRAŞ R&D Center, Körfez, Kocaeli 41790 Türkiye

**Keywords:** Monoelemental calibration solutions, Certified reference material, Characterization, Elemental analysis, Primary difference method, Gravimetric titration

## Abstract

Monoelemental calibration solutions are the most common reference in elemental analysis, linking measurement results to the International System of Units (SI). National Metrology Institutes (NMI) prepare these solutions as certified reference materials (CRM) and determine their elemental mass fraction with high accuracy. Characterization with high accuracy is one of the most critical steps in CRM production. This report compares the approaches taken by the NMIs of Türkiye (TÜBİTAK-UME) and Colombia (INM(CO)) in preparing and characterizing cadmium calibration solutions with a nominal value of 1 g kg^−1^. Each NMI produced CRMs using an independent batch of cadmium calibration solution and assigned mass fraction values to both their own solutions and those of the other NMIs. TÜBİTAK-UME employed a primary difference method (PDM) to assess the purity of a cadmium metal standard, quantifying all possible impurities using a combination of instrumental analytical techniques. The defined purity standard was used for both the gravimetric preparation of the CRM and the calibration of high-performance inductively coupled plasma optical emission spectrometry (ICP-OES) measurements. On the other hand, INM(CO) used gravimetric titration with EDTA to assay cadmium in the CRM solutions. The EDTA salt was previously characterized by titrimetry. Despite the fundamentally different measurement methods and independent metrological traceability paths to the SI, the measurement results exhibited excellent agreement within the stated uncertainties. This comparative analysis demonstrates the effectiveness of varied characterization approaches and underscores the reliability of the cadmium calibration solutions prepared by TÜBİTAK-UME and INM(CO). This collaborative effort highlights the reliability and adaptability of various measurement techniques in fulfilling rigorous metrological standards for elemental calibration solutions. Furthermore, this approach enhances the robustness of the measurements in the field of elemental analysis and contributes traceability to the SI.

**Graphical Abstract:**

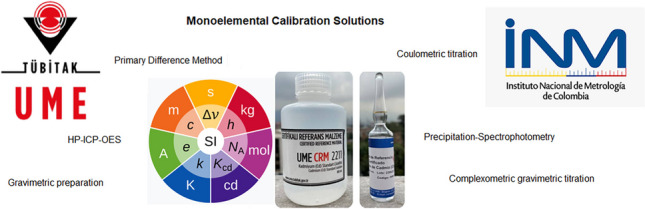

**Supplementary Information:**

The online version contains supplementary material available at 10.1007/s00216-025-05731-4.

## Introduction

Inorganic chemical measurements play a crucial role in addressing various challenges in the environment, healthcare, industry, and technology [1]. Upholding the integrity and reliability of the measurement results requires rigorous metrological traceability to a common reference, ideally the International System of Units (SI), which guarantees their consistency and comparability. The traceability to the SI in inorganic analysis often relies on the analytical calibration of the measurement methods using monoelemental calibration solutions produced as certified reference materials (CRMs) [2, 3]. National Metrology Institutes (NMIs) prepare and characterize these solutions with high accuracy to provide reference mass fraction values with minimal uncertainties. Lower uncertainties in calibration solutions enhance the potential of several instrumental analytical techniques, such as ICP-OES and ICP-MS which are positioned as the most important reference techniques for elemental analysis [4, 5].

The Consultative Committee for Amount of Substance (CCQM) is the maximum authority in chemical metrological traceability, spearheading efforts to harmonize measurement standards globally and fostering collaboration among NMIs to promote SI traceability of chemical and biological measurements [6]. The Inorganic Analysis Working Group of the Consultative Committee for Amount of Substance: Metrology in Chemistry and Biology (CCQM IAWG) has traced important routes to lead NMIs in addressing this task, promoting high comparability and quality among the measurement services worldwide. CCQM IAWG has developed a roadmap for the high-accuracy purity determination of the pure metallic elements used in preparing monoelemental calibration solutions [7]. The alternatives presented in the roadmap fall into two domains: directly assaying the metal using techniques such as titrimetry or coulometry, or determining the metal’s purity indirectly by quantifying and subtracting all possible impurities from an ideal purity value of 1 g g^−1^ [7]. These approaches correspond to the use of classical primary methods (CPM) and primary difference methods (PDM), respectively [4]. The CPMs can be used to assay elemental mass fraction directly in the calibration solutions, while PDMs characterize high-purity metals as primary standard materials, which are then used in the gravimetric preparation of standard solutions at a known mass fraction [7].

The PDM and CPM routes were utilized by the NMIs of Türkiye (TÜBİTAK-UME) and Colombia (INM(CO)), respectively, to characterize cadmium calibration solutions in the CRM production. This report presents a bilateral comparison of the measurement results from both approaches to confirm their metrological compatibility. Cadmium solutions were selected as representative of the transition metals group due to its critical monitoring needs, given its extremely toxic effects on human health [8]. Cadmium is a highly carcinogenic element that causes toxic reactions even at low concentrations and tends to accumulate in the food chain [8]. The determination of cadmium is of special interest in Colombia due to its potential for bioaccumulation by the *Theobroma cacao* plant from which the cocoa beans are harvested, one of the country’s most relevant export commodities [9].

**Fig. 1:**
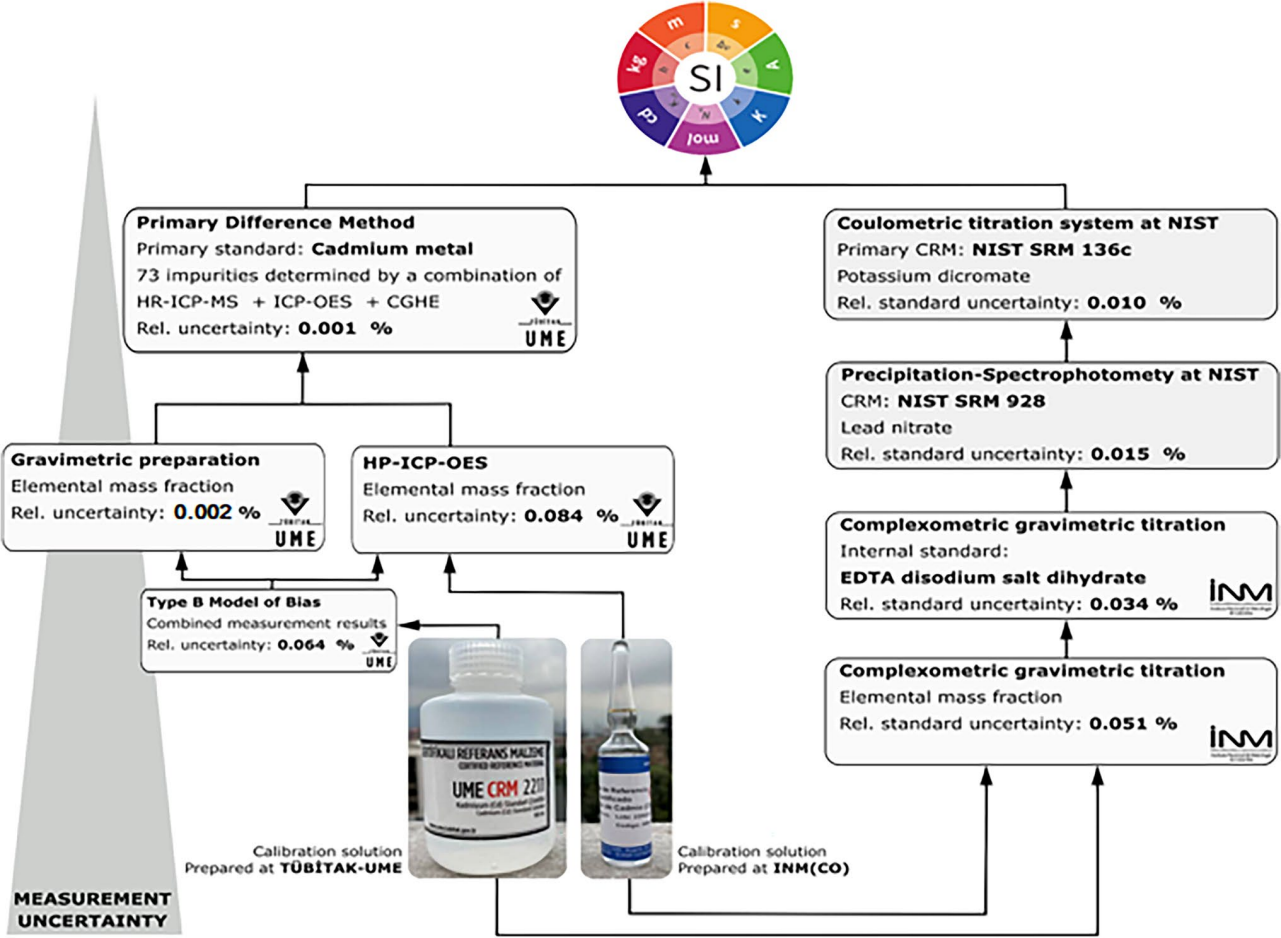
Independent metrological traceability paths used by TÜBİTAK-UME and INM(CO) to link the measurement results for cadmium mass fraction in the calibration solutions to the International System of Units

To compare the characterization approaches, each NMI prepared a batch of cadmium calibration solutions at a nominal mass fraction of 1 g kg^−1^, using independent cadmium sources. They then characterized the cadmium mass fraction in their own solution as well as in the solution prepared by the other NMI. The characterization approach INM(CO) consisted of assaying cadmium in both calibration solutions using direct gravimetric complexometric titration with EDTA On the other hand, TÜBİTAK-UME determined the purity of a cadmium metal standard by measuring the content of 73 impurities and used it in the gravimetric preparation of its cadmium calibration solution at a known mass fraction. Additionally, TÜBİTAK-UME implemented high-performance inductively coupled plasma optical emission spectrometry (HP-ICP-OES) to confirm the gravimetric preparation value of its solution [10] and measure the cadmium mass fraction in the calibration solution prepared at INM(CO). The metrological traceability chains to the SI for the cadmium mass fraction measurement results by both NMIs are illustrated in Fig. 1.

## Experimental methods

### Preparation of the cadmium calibration solutions

Monoelemental calibration solutions are typically prepared under full gravimetric control by dissolving a high-purity metallic portion of the element in acid, and diluting the digest to a predefined elemental mass fraction ranging from 0.1 to 10 g kg^−1^. A small excess of nitric acid is added to enhance the stability of the CRMs. Each NMI prepared a cadmium solution with a nominal mass fraction of 1 g kg^−1^ dissolving pre-weighted cadmium metal in concentrated nitric acid and diluting the digest to the desired concentration with ultrapure water (resistivity > 18 MΩ cm). The nitric acid was purified in-house at each NMI by double sub-boiling distillation starting with acids of the highest commercial purity available (Suprapur®, Merck). INM(CO) used a molded PFA purification system (DST-1000-230, Savillex, USA) while TÜBİTAK-UME used quartz distillation units (duoPUR, Milestone, Italy). The acid was added in excess to a final mass fraction of approximately 2%. The solutions were thoroughly homogenized before packaging.

TÜBİTAK-UME prepared the sample UME-CRM-2211 using granulated, assayed, high-purity cadmium metal (Alfa Aesar, Puratronic, 1–3 mm shot, lot X15E013) and conducted substitution weighing for all steps. The UME-CRM-2211 was aliquoted into 125-mL high-density polyethylene (HDPE) bottles with approximately 100 g solution in each bottle. Conversely, INM(CO) prepared the sample INM-014-1 using high-purity cadmium metal foil (Sigma-Aldrich) pre-cleaned by etching in hydrochloric acid, water, and methanol and dried under argon flow. The INM-014-1 was aliquoted into sealed glass ampoules with a net volume of ca. 10 mL. Both NMIs provided each other with three units of their calibration solutions. The final presentation of the solutions is shown in the pictures at the bottom of Fig. 1.

### Analytical methodology at TÜBİTAK-UME

TÜBİTAK-UME determined the purity of a granulated cadmium metal and certified it as a primary cadmium standard using the PDM described in the “Impurity assessment of high-purity cadmium” section. The high-purity cadmium metal was stored in an argon-filled glove box with controlled humidity and oxygen levels to prevent oxidation. This certified cadmium standard served both as the starting material for the gravimetric preparation of its cadmium monoelemental calibration solution (“Gravimetric preparation” section) and as a traceable calibrant for mass fraction measurements using the HP-ICP-OES method (“HP-ICP-OES measurements for the determination of cadmium mass fraction” section). The uncertainties associated with all measurements were estimated according to the Guide to the Expression of Uncertainty in Measurement (GUM) [11] using the software GUM Workbench [12].

The assigned mass fraction of cadmium in the UME-CRM-2211 calibration solution and its uncertainty were determined by combining gravimetric preparation and HP-ICP-OES measurement results using the Type B Model of Bias (BOB) procedure, which incorporates the bias in the average of the results of the methods as a source of uncertainty [13]. Combining gravimetric values with HP-ICP-OES measurements is a recognized method for assigning values to monoelemental calibration solutions.The HP-ICP-OES method was also used to measure the cadmium mass fraction in solution INM-014-1. The analytical routes chosen by TÜBİTAK-UME are presented on the left side of Fig. 1.

#### Impurity assessment of high-purity cadmium

TÜBİTAK UME followed case 3 of the “Roadmap for the purity determination of pure metallic elements” published by CCQM IAWG, which outlines an impurity assessment approach with expanded measurement uncertainties equal to or less than 0.01% [7, 14]. The impurity assessment approach primarily involves determining and subtracting all potential impurities from 100% [15]. TÜBİTAK-UME developed and validated high-resolution inductively coupled plasma mass spectrometry (HR-ICP-MS), inductively coupled plasma optical emission spectrometry (ICP-OES), and carrier gas hot extraction (CGHE) methods for quantitatively determining elemental impurities in high-purity cadmium metal. The validated methods enabled the quantification of 73 elements in the periodic table, excluding radioactive elements, halogens, and noble gases from the impurity assessment. Impurities present at levels below the corresponding limits of detection (LOD) were considered to have a mass fraction value equal to half the LOD, with expanded relative uncertainties set at 100%.

##### **ICP-OES and HR-ICP-MS measurements for purity determination**

Commercial multi-element standard solutions (HPS, solutions A, B, and C) were used as calibrants for the impurity measurements using ICP-OES (Arcos 2, Spectro, Germany) and HR-ICP-MS (Element 2, Thermo Finnigan, Germany). Solution A contains Al, As, Ba, Be, Bi, B, Cd, Ca, Ce, Cs, Cr, Co, Cu, Dy, Er, Eu, Gd, Ga, Ho, In, Fe, La, Pb, Li, Lu, Mg, Mn, Nd, Ni, P, K, Pr, Re, Rb, Sm, Sc, Se, Na, Sr, Tb, Tl, Th, Tm, U, V, Yb, Y, and Zn in 2% HNO_3_. Solution B contains Sb, Ge, Hf, Mo, Nb, Si, Ag, Ta, Te, Sb, Ti, W, and Zr in 2% HNO_3_ and trace amount of hydrofluoric acid (HF). Solution C contains Au, Ir, Os, Pd, Pt, Rh, and Ru in 15% hydrochloric acid (HCl). NIST Standard Reference Materials (SRM) 3133 and 3181 were used as calibrants for mercury (Hg) and sulfur (S), respectively. All samples and standards were prepared gravimetrically. Analysis of trace impurities in high-purity metals with ICP-MS is subject to matrix effects; hence, standard addition calibration with internal standards (tungsten for Solution A, yttrium for Solutions B and C) was performed to mitigate these effects. Given the rapid exothermic reactions of nitric acid with most metals, perfluoroalkoxy (PFA) tubes (Savillex) were chosen over polypropylene (PP) tubes (Brand) for dissolving cadmium metal due to their greater resistance to hot nitric acid and lower risk of metal leaching from the inner surface.

For HR-ICP-MS measurements, three sets of cadmium stock solutions were prepared for the analysis of elements in solutions A (including Hg and S), B, and C, respectively. Each set consisted of six replicate solutions prepared by dissolving 0.5 g cadmium metal with 2.5 mL HNO_3_ in 15-mL PFA tubes and diluting to a final mass of 10 g with ultrapure water. Before diluting to the final mass, 0.1 mL HF and 0.5 mL HCl were added to the solution sets for elements in solutions B and C, respectively. The standard addition calibration solutions and sample solutions were prepared by diluting the stock cadmium solutions after the addition of internal standard and different spiking levels of the multi-element standard solutions. All calibration standards and sample solutions contained 500 mg kg^−1^ of cadmium and 5 µg kg^−1^ internal standard. Multi-element standard additions were made at 1, 2, 5, 10, 20, and 50 µg kg^−1^. Due to the high-matrix content of the solutions, certain parts of the HR-ICP-MS instrument (sampler cone, skimmer cone, extraction lens, torch, injector, spray chamber, and peristaltic pump tubing) required cleaning after the study. Proper cleaning of the sampler cone, skimmer cone, and extraction lens is critical for HR-ICP-MS after measurements of high Cd-containing samples, as it is necessary to ensure accurate determination of low levels of Cd. The extraction lens in HR-ICP-MS consists of two parts made of graphite, which were soaked in 5% HNO_3_, rinsed with ultrapure water, then dried, and reused. HR-ICP-MS operating conditions for impurity measurements are given in Table S1 (see Electronic Supplementary Material Table S1).

Similarly, for ICP-OES measurements, another three sets of cadmium stock solutions were prepared for the analysis of elements in solutions A (including Hg and S), B, and C, respectively, with three replicates for each set. The solutions were prepared by dissolving 1 g cadmium metal with 5 mL HNO_3_ in 15-mL PFA tubes and diluting to a final mass of 10 g with ultrapure water. Before diluting to the final mass, 0.1 mL HF and 0.5 mL HCl were added to the solution sets for elements in solutions B and C, respectively. The standard addition calibration solutions and sample solutions were prepared by diluting the stock cadmium solutions after the addition of internal standard and different spiking levels of the multi-element standard solutions. All calibration standards and sample solutions contained 10,000 mg kg^−1^ of cadmium and 0.3 mg kg^−1^ internal standard. Multi-element standard additions were made at 10, 20, 50, 100, and 200 µg kg^−1^. ICP-OES measurements were performed in the axial view. In axial view analysis of high-matrix samples, metal ions can accumulate on the surface of the cone and torch. Failure to clean these components thoroughly can lead to arcing during plasma ignition, resulting in torch deformation. Therefore, the cone and torch were cleaned after each sequence. ICP-OES operating conditions for impurity measurements are shown in Table S2 (see Electronic Supplementary Material Table S2).

Due to the lack of reference materials for elemental impurities in high-purity cadmium metal on the market, accuracy testing was performed by adding a known concentration of the standards to dissolved cadmium solutions. For this purpose, 5 µg kg^−1^ and 50 µg kg^−1^ multi-element spiked cadmium solutions were prepared for HR-ICP-MS and ICP-OES, respectively.

##### **CGHE measurements for purity determination**

Measurements of carbon (C), oxygen (O), nitrogen (N), and hydrogen (H) impurities in the high-purity cadmium material were performed by CGHE, also called inert gas fusion. This technique involves melting the sample at high temperatures and measuring the analytes in the gas phase. In the CGHE instrument (Inductar EL Cube, Elementar, Germany), nitrogen is determined with a thermal conductivity detector (TCD), while an electronic hydrogen sensor (EHS) is utilized for hydrogen determination. Carbon and oxygen are first converted to carbon dioxide (CO_2_) and then determined with an infrared detector (IR). The instrument offers two measurement modes: ONH and C modes. The ONH mode allows for simultaneous measurement of O, N, and H, while the C mode is specifically for C measurements. Although switching between modes is not time-consuming, it is discouraged to do it more than once per day to ensure system stabilization, as the instrument requires purging. The CGHE method is commonly employed in the metal and ceramic industries, with limited scientific publications available on C, O, N, and H measurements in high-purity metals using this method [16–18]. Calibration and quality control for O, N, H, and C measurements were performed using the reference materials detailed in Table S3 and Table S4, respectively (see Electronic Supplementary Material Table S3 and Table S4). Since there is no reference material in the market with known amounts of C, O, N, and H in high-purity cadmium metal, the accuracy test was performed using steel reference materials.

#### Gravimetric preparation

TÜBİTAK-UME used metrological weighing to ensure high accuracy of cadmium mass fraction in the gravimetric preparation of UME-CRM-2211. This method involves placing reference standards (R) and test weights (T) on the weighing pan in a cycle R/T/T/R, determining the weighing difference between the test and reference as the average of multiple cycles. 60 g of Cd metal was carefully weighed into a pre-cleaned and pre-weighted 1-L PFA bottle. The weighing differences for the empty PFA bottle and the bottle with cadmium metal were measured by a balance with a mass resolution of 0.1 mg (MSA524S-100-DA, Sartorius AG, Germany). Sub-boiled concentrated nitric acid was then added to the bottle in a sufficient amount to achieve a mass fraction of approximately 2% in the final solution. Upon complete dissolution of the Cd metal, the solution was diluted to 1 L with ultrapure water, ensuring thorough homogenization. Subsequently, the solution was transferred to a pre-cleaned and pre-weighted 60-L HDPE bottle (Kautex, Germany). After rinsing the PFA bottle with ultrapure water several times, the rinse water was added to the HDPE bottle to ensure no residual material was left behind. The HDPE bottle was then filled with ultrapure water to a final mass of 60 kg. To achieve temperature equilibrium, the solution was homogenized in the same laboratory before the final weighing was performed. The weighing differences for the solution and the HDPE bottle were measured using an electromagnetically compensated balance with a mass resolution of 0.1 g (ID5, Mettler Toledo), which was integrated into the automatic weighing system. To ensure accurate measurements, the system was installed on an isolated concrete block to eliminate the transmission of vertical vibrations from the building to the system. Additionally, the system was housed in a plexiglass cabin to reduce airflow and minimize external influences on the measurements. In each measurement, the reference standards and the test weight were entirely removed from the weighing pan, and the balance indicator was zeroed using the zeroing function. For all weighings, OIML E2 class weight standards were utilized as references [19].

Cadmium mass fraction in the CRM solution was calculated using Eqs. 1–3. The symbols used in the equations are given in Table 1.

**Table 1:** The symbols and the definitions used in Eqs. 1 to 3

Symbol	Definition
*w*^*g*^_*Cd*_	Cadmium mass fraction in CRM solution from gravimetric preparation, in g kg^−1^
*m*_*Cd*_	The true mass of the Cd metal, in g
*m*_*Sol*_	The true mass of the CRM solution, in kg
*P*_*Cd*_	Percent purity of the Cd metal, in g g^−1^
*m*_*r*_^*Cd*^	The true mass of the reference standards for Cd weighing, in g
*B*_*r*_^*Cd*^	Air buoyancy factor of the reference standards for Cd weighing, in g g^−1^
*m*_*b*_	The true mass of the PFA bottle, in g
*B*_*b*_	Air buoyancy factor of the PFA bottle, in g g^−1^
*Δm*_*w*_^*Cd*^	The average of R/T/T/R weighing differences for Cd weighing, in g
*B*_*Cd*_	Air buoyancy factor of the Cd metal, in g g^−1^
*m*_*r*_^*Sol*^	The true mass of the reference standard for the CRM solution weighing, in kg
*B*_*r*_^*Sol*^	Air buoyancy factor of the reference standard for the CRM solution weighing, in kg
*m*_*k*_	The true mass of the additional kit, in kg
*B*_*k*_	Air buoyancy factor of the additional kit, in kg kg^−1^
*m*_*c*_	The true mass of the HDPE bottle, in kg
*B*_*c*_	Air buoyancy factor of the HDPE bottle, in kg kg^−1^
*Δm*_*w*_^*Sol*^	The average of R/T/T/R weighing differences for CRM solution weight, in kg
*B*_*Sol*_	Air buoyancy factor of the CRM solution, in kg kg^−1^

1 $${w}_{Cd}^{g}= \frac{{m}_{Cd}}{{m}_{Sol}} \times {P}_{Cd}$$

2 $${m}_{Cd}= \frac{{m}_{r}^{Cd} \times {B}_{r}^{Cd} - {m}_{b} \times {B}_{b}+ {\Delta m}_{w}^{Cd}}{{B}_{Cd}}$$

3 $${m}_{Sol}= \frac{{m}_{r}^{Sol} \times {B}_{r}^{Sol} - {m}_{k} \times {B}_{k}-{m}_{c} \times {B}_{c} + {\Delta m}_{w}^{Sol}}{{B}_{Sol}}$$

#### HP-ICP-OES measurements for the determination of cadmium mass fraction

ICP-OES stands out as one of the most common elemental analysis techniques renowned for its high precision and accuracy. In the determination of cadmium mass fraction in the solutions, the high-performance ICP-OES method (HP-ICP-OES), developed by NIST, was utilized [20–22]. This method incorporates internal standardization to minimize short-term instrumental noise, a drift-correction procedure to minimize instrumental drift, gravimetric sample preparation, and a robust experimental design [23]. Furthermore, to enhance accuracy and reduce measurement uncertainty, Winchester et al. employed an exact matching procedure [24, 25]. This involves meticulously aligning analyte mass fractions and internal standard mass fractions among the calibration standards and sample solutions, ensuring close matching of analyte/IS mass fractions, in addition to matrix compositions. All measurements were conducted using the Spectro Arcos 2 ICP-OES. A crossflow nebulizer with a Scott-type double-pass spray chamber was preferred over a concentric nebulizer with a cyclonic spray chamber to achieve more stable signals. Additionally, a small internal diameter (0.5 mm ID) autosampler probe was chosen to reduce the take-up time and facilitate efficient sample flow. The concentrations of cadmium and the IS (scandium) in the solutions, as well as the measurement wavelength values, were determined based on Salit et al. [20–22]. Signal intensities for 10 mg kg^−1^ Cd and 0.5 mg kg^−1^ Sc were compared at the selected wavelengths to ensure compatibility. The operating conditions for HP-ICP-OES measurements are provided in Table S5 (see Electronic Supplementary Material Table S5).

The instrument underwent a preconditioning period of at least 45 min before the measurement to ensure stability. However, it was observed that the instrumental drift in the initial measurements was larger compared to subsequent ones, independent of the preconditioning time. To mitigate this issue and avoid unstable measurements at the beginning of the sequence, the sequence was halted after the first five measurements and then restarted from the beginning to obtain more stable signals. Autosampler probe rinsing between samples in the sequence was omitted for two reasons: firstly, to reduce the total analysis time, and secondly, to eliminate potential carry-over between the rinse solution and the sample solutions. Given that all solutions in the sequence had similar elemental compositions and mass fractions, the risk of carry-over between solutions was minimized by employing long take-up times. Five independent calibration solutions were gravimetrically prepared from primary Cd standard by dissolving 0.1 g Cd metal with sufficient HNO_3_ to achieve a final mass fraction of approximately 2% HNO_3_ in 125-mL HDPE bottles and diluting to a final volume of 100 mL with ultrapure water. Two 50 g calibration solutions were then prepared from each primary Cd standard by aliquoting 0.5 g into 50-mL PP centrifuge tubes (Brand), spiked with 0.5 g IS stock solution (50 mg kg^−1^ Sc), and diluted with ultrapure water. Similarly, three sub-samples from each of the 3 CRMs and 3 verification solutions were prepared in the same manner as the calibration solutions. To mitigate evaporation effects during sample preparation, the caps of the tubes were kept closed. Buoyancy correction was not applied in the weighings. The prepared calibration, sample, and verification solutions (28 solutions in total) were analyzed using a “randomized complete block sequence” as described by Salit and Turk [22]. This sequence design, crucial for the drift correction procedure—one of the four pillars of the HP-ICP-OES method—ensures each sample is measured once in a random order, repeated up to the desired number of measurements. In this study, each sample was measured six times. Approximately 32 mL of solution was used for six replicates under the given conditions. Since the ICP-OES software (Smart Analyzer Vision) does not allow multiple measurements from a single sample position in the same measurement sequence, the X, Y, and Z coordinates of the autosampler probe had to be redesigned. The analyte-to-internal standard signal ratios produced by ICP-OES were re-evaluated using the drift correction procedure. Cadmium mass fractions in the solutions were calculated using Eqs. 4–8, with the symbols defined in Table 2.

**Table 2:** The symbols and the definitions used in Eqs. 4 to 8

Symbol	Definition
*w*_*Cd*_	Cadmium mass fraction in sample solution, in g kg^−1^
*S*_*SMP*_	Average of the instrument response to the sample solution, in g g^−1^
*S*_*CAL*_	Average of the instrument sensitivity measured with calibration solution, in g mg^−1^
*f*_*CAL*_	Repeatability factor for *S*_*CAL*_
*n*	Number of calibration solutions (*n* = 10)
*R*_*i*_	Drift corrected analyte to internal standard signal ratio for calibration solutions
*m*_*i*_^*IS*^	Mass of internal standard stock solution added to the calibration solutions, in g
*m*_*i*_^*Cd*^	Mass of cadmium element mass fraction in calibration solutions, in mg^−1^
*f*_*SMP*_	Repeatability factor for *S*_*SMP*_
*k*	Number of sample solutions (*k* = 9)
*R*_*j*_	Drift corrected analyte to internal standard signal ratio for sample solutions
*m*_*j*_^*IS*^	Mass of internal standard stock solution added to the sample solutions, in g
*m*_*j*_	Mass of cadmium monoelemental solution in sample solutions, in g

4 $${w}_{Cd}= \frac{{S}_{SMP}}{{S}_{CAL}}$$

5 $${S}_{CAL}= \frac{{\sum }_{i=1}^{n}{S}_{i}}{n} \times {f}_{CAL}$$

6 $${S}_{i}= \frac{{R}_{i} \times {m}_{i}^{IS}}{{m}_{i}^{Cd}}$$

7 $${S}_{SMP}= \frac{{\sum }_{j=1}^{k}{S}_{j}}{k} \times {f}_{SMP}$$

8 $${S}_{j}= \frac{{R}_{j} \times {m}_{j}^{IS}}{{m}_{j}}$$

The accuracy of the measurement results was verified using NIST SRM 3108 Cadmium Standard Reference Material. Three independent solutions were prepared by gravimetrically diluting the NIST SRM 3108 at a cadmium mass fraction of 1 g kg^−1^ and measured using the HP-ICP-OES method on three different days. Three sub-samples from each of the three NIST SRM 3108 solutions were prepared each day for analysis.

### Analytical methodology at INM(CO)

INM(CO) performed complexometric gravimetric titrations using an EDTA salt, with the endpoint indicated potentiometrically via ion-selective electrodes. The methodology consists of two steps: first, characterizing the EDTA salt in terms of the amount of complexing agent concentration, and second, determining the cadmium mass fraction in the sample solutions using EDTA solutions of known concentration. This procedure is similar to the “classical metrological approach” reported by Felber et al. for the complexometric characterization of copper calibration solutions [26]. However, the titration reported by Felber et al. is weight-volumetric, where most of the titrant is added gravimetrically at the beginning and then volumetrically near the endpoint [26]. In contrast, the approach presented here employs gravimetric burettes modified to deliver small portions of titrant solution, allowing for the entire titration to be performed gravimetrically.

The characterization of the EDTA salt utilizes the lead nitrate NIST SRM 928 as the standard for the amount of substance [27]. In summary, standard lead solutions were prepared to a known lead concentration of approximately 4.8 mmol/kg in 2% HNO_3_ and EDTA sample solutions were prepared gravimetrically to an estimated concentration of 10 mmol/kg in ultrapure water. For the EDTA solutions preparation, the disodium dihydrate form of EDTA was dried at 80 °C until constant weight was achieved (Halogen Moisture Analyzer HC103, Mettler Toledo, scale division 0.1 mg), and complete dissolution of the salt was ensured by placing the solutions in an ultrasonic bath for at least 30 min. The gravimetric preparation data relates the EDTA concentration in the titrated samples to the purity of the starting reagent. Air buoyancy correction factors were applied for the gravimetric preparation of solutions. Then, in a single titration, a weighted aliquot of the lead standard solution (approximately 5 g) was placed in a PP titration vessels, and sodium tartrate (240 µmol) and ammonium hydroxide (10 mmol) were added. Ammonium hydroxide creates an alkaline medium, while the sodium tartrate prevents the precipitation of lead hydroxide. The gravimetric burettes were PP syringes with nominal capacity of 5 mL (Precision Dispenser Tips, Brand), adapted with PP capillary tips. The burette was filled with EDTA solution, and its weight was set as the initial tare weight. The mass of added EDTA solution was measured by the difference between the current mass and the initial tare weight. The electric potential difference at a lead selective electrode was continuously monitored under magnetic stirring. Approximately 99.5% of the equivalent EDTA solution mass was added initially, with very small increments made near the endpoint. The endpoint was determined by the steepest change in the potentiometric titration curve. The result is calculated as shown in Eq. 9. The symbols used in the equation are provided in Table 3. The amount of complexing agent concentration is reported as the mass fraction of the dihydrate disodium EDTA salt in the solid reagent.

**Table 3:** The symbols and the definitions used in Eq. 9

Symbol	Definition
*w*_*EDTA*_	Mass fraction of EDTA disodium salt dihydrate in the solid reagent, in g g^−1^
*m*_*SRM928*_	Apparent mass of the NIST SRM used in the preparation of the lead standard solution, in g
*B*_*SRM928*_	Air buoyancy correction factor of the NIST SRM 928, in g g^−1^
*w*_*Pb,SRM928*_	Mass fraction of lead in the NIST SRM 928, in g g^−1^
*m*_*Sol,Pb*_	Apparent total mass of the lead standard solution, in g
*B*_*Sol,Pb*_	Air buoyancy correction factor of the lead standard solution, in g g^−1^
*A*_*Pb,SRM928*_	Lead atomic weight in the NIST SRM 928, in g mol^−1^
*m′*_*aliq*_	Apparent mass of an aliquot of the lead standard solution, in g
*m′*_*eq*_	Apparent equivalent mass of EDTA solution in EDTA characterization titrations, in g
*m′*_*blank*_	Apparent mass of the titration blank in EDTA characterization titrations, in g
*m′*_*EDTA*_	Apparent mass of the EDTA salt used in the preparation of the EDTA solution for EDTA characterization, in g
*B′*_*EDTA*_	Air buoyancy correction factor of the EDTA salt during EDTA characterization, in g g^−1^
*m′*_*Sol,EDTA*_	Apparent total mass of the EDTA solution prepared for EDTA characterization, in g
*B′*_*Sol,EDTA*_	Air buoyancy correction factor of the EDTA solution prepared for EDTA characterization, in g g^−1^
*w*_*EDTA*_	Molecular weight of the EDTA disodium salt dihydrate, in g mol^−1^
*F′*_*rep*_	Repeatability factor of EDTA characterization titrations

9 $${w}_{EDTA}= \frac{{m}_{SRM928}\times {B}_{SRM928}\times {w}_{Pb,SRM928}}{{m}_{Sol,Pb}^\times {B}_{Sol,Pb}\times {A}_{Pb,SRM928}}\times \frac{{m^{\prime}}_{aliq}}{{m^{\prime}}_{eq}-{m^{\prime}}_{blank}}\times \frac{{m^{\prime}}_{Sol,EDTA}\times {B^{\prime}}_{Sol,EDTA}\times {w}_{EDTA}}{{m^{\prime}}_{EDTA}\times {B^{\prime}}_{EDTA}}\times {F^{\prime}}_{rep}$$

A potentially relevant source of uncertainty in the EDTA salt characterization using a lead nitrate SRM arises from the atomic weight of lead, which is used in the measurement model shown in Eq. 9. The reported standard atomic weight of lead spans a broad range due to the natural variability of its isotopic composition [28]. To minimize the impact of this uncertainty on the uncertainty budget, the lead atomic weight applicable to NIST SRM 928 was estimated by measuring the corresponding isotopic ratios using a quadrupole ICP-MS instrument (NexION 300D, Perkin-Elmer, PA, USA). Instrumental isotope fractionation effects were corrected through sample-standard bracketing (SSB) with the lead isotopic standard NIST SRM 981 [27].

After establishing the purity of the EDTA salt in terms of the amount of complexing agent concentration, it can be utilized as a transfer standard for determining the amount of metallic cations in solution. This second titration step follows a similar procedure to the EDTA characterization described earlier, using a cadmium-selective combination electrode instead of the lead-selective electrode. The cadmium mass fraction in the solution is calculated as shown in [4]. The symbols used in Eq. 10 are provided in Table 4. All uncertainty calculations were performed according to the GUM using the R package propagate [29].

**Table 4:** The symbols and the definitions used in Eq. 10

Symbol	Definition
*w*_*Cd*_	Cadmium mass fraction in the cadmium sample solution, in g kg^−−1^
*m*_*EDTA*_	Apparent mass of the EDTA salt for an EDTA solution used in cadmium titration, in g
*B*_*EDTA*_	Air buoyancy correction factor of the EDTA salt, used in cadmium titrations, in g g^−1^
*m*_*Sol,EDTA*_	Apparent total mass of the EDTA solution prepared for cadmium, in g
*B*_*Sol,EDTA*_	Air buoyancy correction factor of the EDTA solution prepared for cadmium titrations, in g g^−1^
*m*_*eq*_	Apparent equivalent mass of the EDTA solution prepared for cadmium titrations, in g
*m*_*blank*_	Apparent mass of a titration blank in cadmium titrations, in g
*m*_*aliq*_	Apparent mass of an aliquot of cadmium sample solution, in g
*A°*_*Cd*_	Cadmium standard atomic weight, in g mol^−1^
*F*_*rep*_	Repeatability factor of cadmium titrations
*δ*_*imp*_	Error term to account for possible impurities in the cadmium calibration solution, in g kg^−1^
*δ*_*FC*_	Error term to account for the difference of EDTA binding constants to cadmium and lead, in g kg^−1^

10 $${w}_{Cd}=\frac{{w}_{EDTA}\times {m}_{EDTA} \times {B}_{EDTA}}{{m}_{Sol,EDTA}\times {B}_{Sol,EDTA}\times {W}_{EDTA}}\times \frac{{m}_{eq}-{m}_{blank}}{{m}_{aliq}}\times {A}_{Cd}^{0}\times {F}_{rep}\times {10}^{3} +{\delta }_{imp}+{\delta }_{FC}$$

Accuracy of the cadmium mass fraction titration results was verified using the NIST SRM 3108 cadmium standard solution. Standard solutions were prepared by gravimetrically diluting NIST SRM 3108 to a cadmium mass fraction close to 1 g kg^−1^ in 2% HNO_3_ and titrated alongside the samples. Between two and four replicate titrations were performed for the solutions on four different days.

## Results

### Purity assessment of cadmium metal in TÜBİTAK-UME

The LODs, recoveries, and typical correlation coefficients attained for the determination of 69 impurities using ICP-OES and HR-ICP-MS are presented in Table S6 (see Electronic Supplementary Material Table S6). The recovery values reflect that standard addition calibration with internal standardization deemed useful in achieving good accuracy for the ICP-based methods.

The samples containing 20 times more cadmium could be aspirated to ICP-OES compared to HR-ICP-MS due to differences in their respective sensitivities and capabilities. When the solutions contain 3 or 4 times more Cd than 0.05%, metal ions start to accumulate on the sampler cone, resulting in a rapid decrease in signal intensity during HR-ICP-MS measurements. Studies by Pattberg and Matschat showed an approximately 50% decrease in signal intensity of a 10 µg L^−1^ Y solution at 0.2% Cu matrix concentration [30]. Similarly, Karandashev et al. reported a sensitivity decrease by a factor of 1.5 after only 3–4 solutions had been measured for samples containing 0.08% tungsten, while the signal change was negligible in samples containing 0.08% sodium [31]. To maintain stability, the matrix concentration was kept at 0.05% Cd for HR-ICP-MS measurements. This ensured that the signal intensity of the 5 µg kg^−1^ W (internal standard) remained stable throughout the 3-h measurement period, during which 25 solutions were analyzed (see Electronic Supplementary Material Fig. S1).

The LODs, recoveries, and typical correlation coefficients attained for the determination of four impurities (O, N, H, and C) using CGHE are presented in Table S7 (see Electronic Supplementary Material Table S7).

The LODs, recoveries, and correlation coefficients for the three analytical techniques were suitable for assessing cadmium purity using the PDM. Out of the 73 measured elements, only six (Ag, Cu, Ge, Ni, S, and C) had mean concentrations exceeding the LODs, with a total concentration of 14.76 mg kg^−1^ in mass fraction. The purity value for the cadmium primary standard was determined to be 99.99526%, with an expanded uncertainty of 0.00232% (*k* = 2).

### Cadmium mass fraction determinations by TÜBİTAK-UME

The results of the accuracy test of HP-ICP-OES method using the NIST SRM 3108 are presented in Table 5. The recovery values and the relative standard deviations (RSD) are satisfactory, ranging from 100.04 to 100.08% and from 0.06 to 0.07%, respectively. Accuracy verification is crucial, particularly when the same high-purity cadmium material is used for both the gravimetric preparation of the cadmium monoelemental solution and the calibration of the ICP-OES instrument in the HP methodology. The maximum relative bias for day-averaged results was 0.08%, which is smaller than the relative expanded uncertainty of the certified cadmium mass fraction in the NIST SRM 3108 (0.19%, *k* = 2.23). These results indicate no significant bias, confirming that the measurements were accurate and precise within acceptable limits, thereby validating the reliability of the HP-ICP-OES method for determining the cadmium mass fraction in monoelemental calibration solutions.

**Table 5:** The accuracy study results for HP-ICP-OES measurements at TÜBİTAK-UME

Solution No.	Recovery (%)		
	Day 1	Day 2	Day 3
1	100.12	100.14	100.10
2	100.04	100.00	99.98
3	100.01	100.10	100.02
Mean	100.06	100.08	100.04
RSD	0.06	0.07	0.06

The measurement results for the cadmium mass fraction in the monoelemental calibration solutions obtained at TÜBİTAK-UME are presented in Table 6. These cadmium mass fraction values are further compared to the results obtained by the analytical method implemented at INM(CO) in the “Comparison of the measurement results by the different approaches” section.

**Table 6:** TÜBİTAK-UME measurement results for cadmium mass fraction in the calibration solutions, by combination of HP-ICP-OES with gravimetric preparation (solution UME-CRM-2211), and by HP-ICP-OES (solution INM–014-1)

Sample solution	Cadmium mass fraction		
	Mean (g kg^−1^)	Expanded uncertainty (*k* = 2) (g kg^−1^)	Relative standard uncertainty
UME-CRM-2211	0.99973	0.00129	0.064%
INM-014-1	1.00070	0.00152	0.076%

Table 7 identifies and quantifies the principal sources of uncertainty in measurements conducted by TÜBİTAK-UME using HP-ICP-OES. The uncertainty contributions are given as percentages for two reference materials: INM-014-1 and UME-CRM-2211. The most significant source of uncertainty is instrument sensitivity for calibration solutions (S_CAL_), contributing 42.6% for INM-014-1 and 48.3% for UME-CRM-2211. The second biggest uncertainty source is repeatability factor for S_CAL_ (f_CAL_), contributing 24.9% for INM-014-1 and 37.9% for UME-CRM-2211. The uncertainties of *S*_*CAL*_ and *f*_*CAL*_ are related to the similarity (exact matching) of the concentrations of the prepared calibration solutions. It has been observed that the closer the concentrations of the calibration solutions, the lower their uncertainty.

**Table 7:** Main sources in the uncertainty of TÜBİTAK-UME measurement results using HP-ICP-OES method

Uncertainty source		Relative contribution (%)	
		INM-014-1	UME-CRM-2211
*S*_*CAL*_	Instrument sensitivity for calibration solutions	42.6	48.3
*f*_*CAL*_	Repeatability factor for *S*_*CAL*_	24.9	37.9
*f*_*SMP*_	Repeatability factor for *S*_*SMP*_	15.4	5.9
*m*	Weighing	3.8	3.0
*Others*		13.3	5.9

The relative standard uncertainties of the measurement results are below the 0.1% uncertainty threshold expected for monoelemental calibration solutions. The measurement results indicate that the calibration solutions were prepared with minor, statistically insignificant deviations from the nominal cadmium mass fraction value of 1 g kg^−1^.

### Cadmium mass fraction determinations by INM(CO)

The results of the accuracy test for the gravimetric complexometric titration method using NIST SRM 3108 are presented in Table 8. The recovery values are satisfactory, ranging from 99.92 to 99.98%. The RSD of the first day is 0.03%. The maximum relative bias for day-averaged results is −0.08%, which is smaller in absolute value than the relative expanded uncertainty of the certified cadmium mass fraction in the NIST SRM 3108. This provides evidence that there is no significant bias, and the measurements were accurate and precise within acceptable limits. However, there seems to be a systematic underestimation of the measurand by the titration method.

**Table 8:** The accuracy study results for titration measurements at INM(CO)

Titration No.	Recovery (%)			
	Day 1	Day 2	Day 3	Day 4
1	99.96	99.96	99.98	99.99
2	99.91	99.96	99.94	99.98
3	99.94	-	-	-
4	99.90	-	-	-
Mean	99.92	99.96	99.96	99.98
RSD	0.03	-	-	-

The absence of significant bias for the measurement results of the cadmium standard solutions presented in Table 8 demonstrates that an EDTA salt characterized using a lead nitrate CRM is suitable for measuring cadmium mass fraction in the calibration CRMs. This is a consequence of the binding constants of EDTA with lead and cadmium ions being similar. The EDTA complex formation constants with lead and cadmium differ in their base-10 logarithms by less than 10% [32], indicating that the binding of the chelate to the metal ions occurs to a comparable extent. The error term reflecting the difference in the binding constants (${\delta }_{FC}$) is not significative, as the amount of unchelated lead or cadmium ions at the titration endpoint is minimal in alkaline media.

The measurement results obtained at INM(CO) for the cadmium mass fraction in the calibration solutions are presented in Table 9. These values are further compared to the measurement results obtained by the analytical method implemented by TÜBİTAK-UME in the “Comparison of the measurement results by the different approaches” section. The main sources of uncertainty and their relative contributions of INM(CO) titration measurement results are presented in Table 10.

**Table 9:** INM(CO) measurement results for cadmium mass fraction in the two monoelemental solutions by gravimetric complexometric titration

Sample solution	Cadmium mass fraction		
	Mean (g kg^−1^)	Expanded uncertainty (*k* = 2) (g kg^−1^)	Relative standard uncertainty
UME-CRM-2211	0.99937	0.00094	0.047%
INM-014-1	1.00061	0.00101	0.051%

**Table 10:** Main sources in the uncertainty of INM(CO) measurement results using gravimetric complexometric titration

Uncertainty source		Relative contribution (%)	
		INM-014-1	UME-CRM-2211
*F*_*rep*_	Repeatability of cadmium solution titrations	28.7	9.0
*w*_Pb_	Mass fraction of lead in the NIST SRM 928	23.6	27.5
*m*_*blank*_	Mass of a titration blank in cadmium titrations	13.3	19.7
*m*′_*blank*_	Mass of a titration blank during EDTA characterization	11.3	13.1
*F*′_*rep*_	Repeatability of EDTA characterization titrations	16.9	8.1
*m*′_EDTA_	Apparent mass of EDTA salt during EDTA characterization	6.0	7.0
*m*_*SRM*928_	Apparent mass of NIST SRM 928 for the lead standard solution	4.1	6.7
${\delta }_{imp}$	Error term due to possible impurities present in the CRMs	2.7	3.1
*Others*		3.5	5.8

Repeatability of cadmium titrations (*F*_*rep*_) is the largest source of uncertainty for INM-014-1 (28.7%) and a significant source for UME-CRM-2211 (9.0%). On the other hand, the mass fraction of lead in the NIST SRM 928 (*w*_*Pb*_) is the another significant uncertainty source for both, contributing 23.6% for INM-014-1 and 27.5% for UME-CRM-2211. Analogously to the measurement results reported by TÜBİTAK-UME (“Cadmium mass fraction determinations by TÜBİTAK-UME” section), the relative standard uncertainties are smaller than the 0.1% uncertainty level expected for monoelemental calibration solutions.

### Comparison of the measurement results by the different approaches

**Fig. 2:**
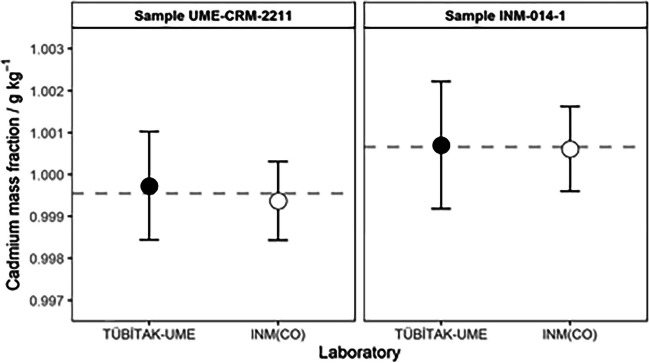
Cadmium mass fraction measurement results from TÜBİTAK-UME and INM(CO) for the calibration solutions UME-CRM-2211 and INM-014-1. Error bars represent expanded uncertainties with a coverage factor *k* = 2. Dashed lines indicate the mean results of each solution

The measurement results obtained by TÜBİTAK-UME (formerly presented in Table 6) and INM(CO) (formerly presented in Table 9) for the two cadmium calibration solutions are shown graphically in Fig. 2. Both solutions were prepared with cadmium mass fractions close to the target nominal value of 1 g kg^−1^, and the individual measurement results from both NMIs showed relative differences smaller than 0.036%. The overlapping error bars shown in Fig. 2 suggest that the measurement results are compatible within stated measurement uncertainties. However, in both cases, the results reported by TÜBİTAK-UME are slightly larger than those reported by INM(CO). This behavior is consistent with the outcomes of the accuracy tests performed for the HP-ICP-MS method and the titration method, as presented in Tables 5 and 8, respectively.

The relative differences between the individual measurement results are 0.036% for UME-CRM-2211 and 0.009% for INM-014-1. These small differences can be assessed for statistical significance within stated uncertainties regarding their metrological compatibility [33]. Two results, *x*_*1*_ and *x*_*2*_, with standard uncertainties *u(x*_*1*_*)* and *u(x*_*2*_*)*, respectively, are said to be metrologically compatible if the absolute value of their difference is smaller than the expanded uncertainty of that difference, using a coverage factor *k*, as presented in Eq. 11. Table 11 presents the metrological compatibility expressions for the samples analyzed.

**Table 11:** Metrological compatibility of the measurement results

Sample	$\left|{x}_{1}- {x}_{2}\right|$(g kg^−1^)	$k \times \sqrt{{u}^{2}({x}_{1})+{u}^{2}({x}_{2})}$(g kg^−1^)	Metrological compatibility
UME-CRM-2211	0.00036	0.00160	Yes
INM-014-1	0.00009	0.00182	Yes

11 $$\left|{x}_{1}-{x}_{2}\right| \le k \times \sqrt{{u}^{2}({x}_{1})+{u}^{2}({x}_{2})}$$

The results from the NMIs are metrologically compatible, providing evidence of their traceability to the SI. This implies that the analytical methods were correctly implemented and the uncertainties were reasonably estimated. The uncertainties reported by INM(CO) are smaller than those reported by TÜBİTAK-UME; nevertheless, this difference is small. It is important to consider that the EDTA titrimetry is not selective to cadmium ions, hence the presence of impurities shall be assessed in the CRMs and considered in the measurement model for uncertainty calculations. The use of high-purity nitric acid and ultrapure water is essential for minimizing the impurities in the calibration solutions and reducing their potential impact on the measurement results, but the potential impurities must be quantified by using an analytical technique suitable for trace analysis like ICP-MS. Tables 7 and 10 provide a detailed breakdown of the factors that significantly impact the uncertainty in the different analytical measurements applied to the same materials. Understanding these contributions is crucial for improving measurement accuracy and precision. As high-purity chemicals were used in the preparation of the CRMs, the impact of potential impurities in the CRMs is insignificant for EDTA titrimetry.

Preparation and characterization of calibration solutions are core capabilities that NMIs demonstrate formally through participation in official comparisons organized under the framework of the Mutual Recognition Agreement of the International Committee for Weights and Measures (CIPM MRA). To date, three official comparisons have served to benchmark the NMIs capabilities in monoelemental calibration solutions: CCQM-K8, which tested aluminum, copper, magnesium, and iron in 1999 [34]; CCQM-K87, which tested chromium, cobalt and lead in 2010 [35]; and CCQM-K143, which focused on copper in 2017 [36]. Notably, only CCQM-K143 specifically assessed the gravimetric preparation of these solutions. TÜBİTAK-UME achieved partially satisfactory results in CCQM-K87 and performed excellently in CCQM-K143, while INM(CO) was not ready to participate at that time. The comparison of the analytical approaches presented in this report served TÜBİTAK-UME to confirm its capabilities in the preparation and characterization of calibration solutions, while also enabling INM(CO) to preliminary assess its capabilities in preparation for upcoming CCQM official comparisons.

## Conclusions

Different measurement principles were applied to characterize independent monoelemental cadmium solutions prepared at a nominal value of 1 g g^−1^. This exercise tested the preparation and elemental mass fraction measurement capabilities of cadmium monoelemental calibration solutions. TÜBİTAK-UME determined the cadmium mass fraction in sample UME-CRM-2211 by averaging the results of gravimetric preparation and HP-ICP-OES method, achieving standard relative uncertainties of 0.064%. For sample INM-014-1, the cadmium mass fraction was determined solely using HP-ICP-OES, with a standard relative uncertainty of 0.076%. In contrast, INM(CO) employed complexometric potentiometric titrimetry for determining the cadmium mass fraction in the monoelemental calibration solutions, attaining standard relative uncertainties between 0.047 and 0.051%.

The methods used by the NMIs are based on distinct measurement principles, each following independent metrological traceability chains to the SI. The reported results demonstrate excellent agreement in terms of their metrological compatibility within the specified measurement uncertainties. TÜBİTAK-UME utilized the PDM to accurately determine the purity of the cadmium metal, which can be further used as a higher-hierarchy primary standard for amount of substance. The CPM implemented by INM(CO) is constrained to assaying cadmium in high-purity solutions without interfering elements. The monoelemental solutions prepared by each institute closely adhered to the target nominal value of 1 g kg^−1^ with negligible deviations. The relative standard uncertainties of the characterization results met the expected levels for monoelemental calibration solutions characterized at a metrological level.

The metrological equivalence of the measurement results underscores the robustness and reliability of the different characterization approaches for the characterization of monoelemental calibration solutions. This collaborative effort highlights the reliability and adaptability of various measurement techniques in meeting rigorous metrological standards for these CRMs. The characterization methods presented in this study are prepared for testing their performance in upcoming official comparisons focusing on the preparation of monoelemental calibration solutions.

## Supplementary Information

Below is the link to the electronic supplementary material.

Supplementary file1 (DOCX 112 KB)
