# Supplementary material for: Comparison of different characterization approaches for monoelemental calibration solutions at two national metrology institutes
Source: Anal Bioanal Chem. 2025 Jan 16;417(12):2603–16. doi: 10.1007/s00216-025-05731-4 (PMC12003560; doi:10.1007/s00216-025-05731-4)
Supplement: Supplementary file 1 — Supplementary file1 (DOCX 112 KB) [file 216_2025_5731_MOESM1_ESM.docx]

**Supplementary information of**

**Comparison of different characterization approaches for monoelemental calibration solutions at two national metrology institutes**

## **Murat Tunç^1*^, Cristhian Paredes^2^, F. Gonca Coşkun^1^, Juliana Serna^2^, Merve Caner^3^**

## ^1^ TÜBİTAK-UME National Metrology Institute, Gebze Yerleşkesi Barış Mah. Dr. Zeki Acar Cad. No:1, 41470, Gebze, Kocaeli Türkiye.

## ^2^ Instituto Nacional de Metrología de Colombia, Av Carrera 50 no 26 - 55 Int. 2, 111321, Bogotá, Colombia.

## ^3^ Turkish Petroleum Refineries Co. TÜPRAŞ R&D Center, 41790, Körfez, Kocaeli, Türkiye.

## * Corresponding author

This supplementary material contains one figure and seven tables outlining instrumental parameters, reference materials and performance characteristics of the methods used in this study.

***Fig. S1*** The signal intensity of 5 µg kg-1 W (IS) solution at a matrix concentration of 0.05 % Cd throughout 3 hours of measurements (25 solutions)


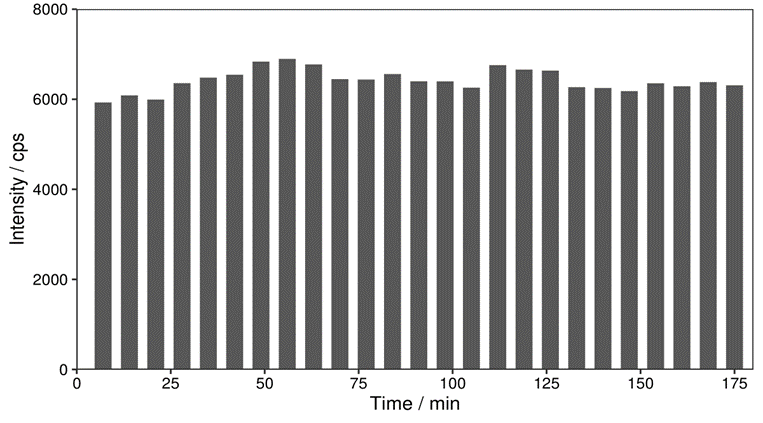


**Table S1.** HR-ICP-MS operating conditions for impurity measurements in cadmium metal

| Parameter | Value |
| --- | --- |
| Plasma gas flow rate (Ar) | 15 L min^−1^ |
| Auxiliary gas flow rate (Ar) | 0.8 L min^−1^ |
| Nebulizer gas flow rate (Ar) | [1.1 - 1.2] L min^−1^ (optimized daily) |
| RF power | 1400 W |
| Spray chamber type | Cyclonic, PFA (ESI) |
| Nebulizer type | OpalMist, PFA (Glass Expansion) |
| Resolution mode | High |
| Runs/Passes | 3/3 |
| Sample time | 0.01 s |
| Sample per peak | 30 |
| Search window | 100 |
| Scan type | Escan |
| Detection mode | Both |
| Cones | Nickel |
| Take up time | 120 s |
| Rinse time | 40 s |

**Table S2.** ICP-OES operating conditions for impurity measurements in cadmium metal

| Parameter | Value |
| --- | --- |
| Plasma gas flow rate (Ar) | 14.5 L min^−1^ |
| Auxiliary gas flow rate (Ar) | 0.8 L min^−1^ |
| Nebulizer gas flow rate (Ar) | 0.85 L min^−1^ |
| RF power | 1600 W |
| Spray chamber type | Cyclonic, PFA (Spectro) |
| Nebulizer type | Mira Mist, Teflon (Burgener) |
| Torch position | End on (axial) view |
| Peristaltic pump speed | 30 rpm |
| Sample uptake rate | 2 mL min^−1^ |
| Take-up time | 45 s |
| Data transport | Complete spectrum |
| Measurement time | 144 s |
| Integration time | 48 ms (best SNR mode) |
| Replicate | 3 |

**Table S3.** Reference materials used for oxygen, nitrogen, and hydrogen calibration of the CGHE method

| Company | Part No. | Lot No. | *w*_O_ / mg kg^−1^ | *w*_N_ / mg kg^−1^ | *w*_H_ / mg kg^−1^ |
| --- | --- | --- | --- | --- | --- |
| NIST | 360b | - | 1430 | 45 | 16.01 |
| NIST | 2453a | - | - | - | 125 |
| Alpha Resources | AR675 | 514C | 580 | 3830 | - |
| Alpha Resources | AR1650 | 519C | 100 | 102 | - |
| Alpha Resources | AR675 | 1018b | 1580 | 2250 | - |
| Brammer Standard | BS HON U | - | 23 | 2540 | 1 |
| Leco | 502-903 | 0673 | 40 | 509 | 4.4 |
| Leco | 502-855 | 0718 | 121 | 673 | 6.7 |
| Leco | 502-913 | 0720 | 66 | 557 | 3.5 |

**Table S4.** Reference materials used for carbon calibration of the CGHE method

| Company | Part No. | Lot No. | *w*_C_ / mg kg^−1^ |
| --- | --- | --- | --- |
| ECISS | 285-2 | - | 18 |
| ECISS | 058-2 | - | 4240 |
| EURONORM | 031-3 | - | 550 |
| Leco | 501-675 | J0282-9 | 690 |
| Leco | 501-679 | J0386-30 | 8960 |

**Table S5.** ICP-OES operating conditions for high-performance cadmium measurements

| Parameter | Value |
| --- | --- |
| Plasma gas flow rate (Ar) | 12 L min^-1^ |
| Auxiliary gas flow rate (Ar) | 1 L min^-1^ |
| Nebulizer gas flow rate (Ar) | 0.85 L min^-1^ |
| RF power | 1400 W |
| Spray chamber type | Scott double pass |
| Nebulizer type | Crossflow |
| Autosampler probe ID | 0.5 mm |
| Torch position | Side on (radial) view |
| Peristaltic pump speed | 30 rpm |
| Sample uptake rate | 2 mL min^-1^ |
| Take-up time | 90 s |
| Data transport | Regions of interest |
| Measurement time | 100 s |
| Integration time | 100 ms |
| Replicate | 10 |
| Analyte (Cd) wavelength | 226.502 nm |
| Internal standard (Sc) wavelength | 361.382 nm |

**Table S6.** Detection limits (LOD), recoveries (Rec.), and correlation coefficient (R^2^) for ICP-OES and HR-ICP-MS impurities measurements in cadmium metal

ICP-OES HR-ICP-MS

Element Wavelength (nm) LOD (mg kg^−1^) Rec. (%) R^2^ Isotope LOD ( mg kg^−1^) Rec. (%) R^2^

Au - - - - 197Au 0.537 82 0.9991

Ag 328.068 0.038 99 0.9999 107Ag 0.097 98 1.0000

Al 396.152 0.081 102 0.9999 27Al 0.357 111 0.9997

As 189.042 0.166 102 0.9999 75As 0.284 99 0.9998

B 182.641 0.056 102 1.0000 11B 0.529 100 1.0000

Ba 455.404 0.025 107 0.9987 137Ba 0.223 96 0.9999

Be 234.861 0.016 104 0.9994 9Be 0.624 98 0.9998

Bi 190.241 0.401 105 0.9998 209Bi 0.058 93 0.9995

Ca 315.887 0.553 103 1.0000 44Ca 1.203 109 0.9997

Ce 413.380 0.093 102 1.0000 140Ce 0.182 99 0.9998

Co 238.892 0.045 106 0.9993 59Co 1.173 97 0.9987

Cr 284.984 0.046 103 0.9998 52Cr 0.354 112 0.9996

Cs - - - - 133Cs 0.132 108 0.9994

Cu 324.754 0.044 102 1.0000 63Cu 0.192 97 0.9997

Dy 353.170 0.057 101 1.0000 163Dy 0.221 99 0.9999

Er 337.271 0.047 105 0.9999 166Er 0.134 100 0.9999

Eu 381.967 0.019 105 0.9999 153Eu 0.132 104 0.9996

Fe 259.941 0.033 105 0.9998 56Fe 1.444 115 1.0000

Ga 141.444 0.045 102 0.9999 69Ga 0.173 107 0.9996

Gd 342.247 0.039 102 1.0000 157Gd 0.117 99 0.9999

Ge 164.919 0.058 100 0.9999 72Ge 0.094 101 0.9995

Hf 264.141 0.213 98 1.0000 178Hf 0.135 92 0.9998

Hg - - - - 202Hg 0.253 88 0.9990

Ho 345.600 0.069 101 1.0000 165Ho 0.132 98 0.9999

In 303.936 0.057 95 0.9993 115In 0.468 92 0.9999

Ir 212.681 0.638 99 0.9999 193Ir 0.168 96 1.0000

K 766.491 0.554 100 0.9999 - - - -

La 379.478 0.053 101 1.0000 139La 0.190 97 0.9997

Li 670.780 0.045 104 0.9996 7Li 0.302 104 0.9999

Lu 261.542 0.034 101 1.0000 175Lu 0.089 98 0.9999

Mg 279.553 0.058 105 0.9998 24Mg 0.322 97 0.9995

Mn 294.921 0.027 102 1.0000 55Mn 0.325 97 0.9995

Mo 203.909 0.044 99 0.9999 98Mo 0.102 96 0.9996

Na 589.592 0.146 102 0.9998 - - - -

Nb 309.418 0.042 99 0.9999 93Nb 0.094 99 0.9992

Nd 401.225 0.114 102 0.9999 146Nd 0.308 93 0.9991

Ni 231.604 0.059 102 0.9999 60Ni 0.350 112 0.9997

Os 189.863 0.149 98 0.9999 189Os 0.337 94 0.9996

P 177.495 0.067 101 1.0000 31P 0.648 91 0.9980

Pb 168.215 0.097 103 0.9999 208Pb 0.211 93 0.9990

Pd 324.270 0.459 103 0.9996 104Pd 0.235 95 0.9997

Pr 414.311 0.122 102 1.0000 141Pr 0.198 96 0.9996

Pt 177.708 0.110 101 1.0000 195Pt 0.349 98 0.9994

Rb - - - - 85Rb 0.178 111 0.9987

Re 227.525 0.469 98 0.9997 185Re 0.232 96 0.9995

Rh 343.489 0.199 99 1.0000 103Rh 0.120 102 1.0000

Ru 240.272 0.089 99 0.9999 101Ru 0.140 103 0.9997

S 180.731 0.099 103 0.9999 32S 1.177 117 0.9993

Sb 206.833 0.225 99 0.9997 121Sb 0.257 93 0.9998

Sc 364.279 0.015 105 0.9998 45Sc 0.194 106 0.9995

Se 196.090 0.264 104 0.9998 77Se 0.814 96 0.9989

Si - - - - 28Si 2.076 95 0.9999

Sm 359.260 0.178 100 1.0000 147Sm 0.189 92 0.9993

Sn 175.790 0.045 98 0.9999 118Sn 0.255 90 0.9997

Sr 421.552 0.027 105 0.9999 88Sr 0.111 96 0.9997

Ta 240.063 0.131 99 0.9998 181Ta 0.149 91 0.9998

Tb 350.917 0.093 102 1.0000 159Tb 0.133 99 1.0000

Te 170.000 0.202 97 0.9999 125Te 0.345 90 0.9994

Th 401.913 0.276 104 0.9999 232Th 0.110 106 0.9998

Ti 336.121 0.052 99 0.9999 47Ti 0.744 85 0.9999

Tl 132.171 0.089 101 1.0000 205Tl 0.121 94 0.9995

Tm 342.508 0.055 102 1.0000 169Tm 0.138 100 0.9999

U 385.958 0.808 116 0.9992 238U 0.175 98 0.9997

V 309.311 0.031 102 1.0000 51V 0.204 94 0.9995

W 220.448 0.393 99 0.9997 182W 0.175 91 0.9998

Y 377.433 0.021 106 0.9998 89Y 0.149 95 0.9993

Yb 328.937 0.020 105 0.9995 172Yb 0.125 100 1.0000

Zn 202.613 0.060 101 1.0000 66Zn 0.728 93 0.9990

Zr 339.198 0.055 100 0.9996 90Zr 0.167 90 0.9996

The correlation coefficient (R^2^) is an indicator of linearity for the calibration curve. Data obtained R^2^ > 0.995 means the linearity of the calibration curve is fit for the purpose.

**Table S7.** Detection limits (LOD), recoveries and correlation coefficient (R^2^) for CGHE measurements

| Element | LOD (mg kg^−1^) | Recovery (%) | *R*^2^ |
| --- | --- | --- | --- |
| O | 15 | 108 | 0.9977 |
| N | 40 | 101 | 0.9982 |
| H | 0.6 | 98 | 0.9978 |
| C | 4.5 | 107 | 0.9998 |

The correlation coefficient (R^2^) is an indicator of linearity for the calibration curve. Data obtained R^2^ > 0.995 means the linearity of the calibration curve is fit for the purpose
